# Interactions between *DRD2/ANKK1* TaqIA Polymorphism and Dietary Factors Influence Plasma Triglyceride Concentrations in Diabetic Patients from Western Mexico: A Cross-sectional Study

**DOI:** 10.3390/nu11122863

**Published:** 2019-11-22

**Authors:** Omar Ramos-Lopez, Roberto Mejia-Godoy, Kevin J. Frías-Delgadillo, Rafael Torres-Valadez, Aurelio Flores-García, Sergio Sánchez-Enríquez, Pedro Aguiar-García, Erika Martínez-López, Eloy A. Zepeda-Carrillo

**Affiliations:** 1 Facultad de Medicina y Psicología, Universidad Autónoma de Baja California, Tijuana, Baja California 22427, Mexico; 2 Unidad Especializada en Investigación, Desarrollo e Innovación en Medicina Genómica, Centro Nayarita de Innovación y Transferencia de Tecnología, Universidad Autónoma de Nayarit, Tepic, Nayarit 63155, Mexico; robertoto_mejia994@hotmail.com (R.M.-G.); queviin.bay@hotmail.com (K.J.F.-D.); forever_genesis_08@hotmail.com (R.T.-V.); 3 Unidad Académica de Medicina, Laboratorio de Investigación en Enfermedades Crónico-Degenerativas, Universidad Autónoma de Nayarit, Tepic, Nayarit 63155, Mexico; affloresg@gmail.com (A.F.-G.); pagarcia_55@yahoo.com (P.A.-G.); 4 Departmento de Biología Molecular y Genómica, Centro Universitario de Ciencias de la Salud, Universidad de Guadalajara, Guadalajara, Jalisco 44340, Mexico; serlucis@hotmail.com (S.S.-E.); erikamtz27@yahoo.com.mx (E.M.-L.); 5 Hospital Civil Dr Antonio González Guevara, Servicios de Salud en Nayarit, Tepic, Nayarit 63000, Mexico

**Keywords:** diabetes, *DRD2/ANKK1* TaqIA polymorphism, diet, triglycerides, personalized nutrition

## Abstract

This study aimed to screen relevant interactions between *DRD2/ANKK1* TaqIA polymorphism and dietary intakes with reference to phenotypical features in patients with T2D from western Mexico. In this cross-sectional study, a total of 175 T2D patients were enrolled. Dietary intake was evaluated using 3-day food records and appropriate software. Glycemic and blood lipid profiles were measured by standardized methods. Genotyping of the *DRD2/ANKK1* TaqIA polymorphism was performed by the RFLP method. Gene-diet interactions regarding anthropometric and metabolic phenotypes were screened by adjusted multiple linear regression analyses. Genotype frequencies of the *DRD2/ANKK1* TaqIA polymorphism were A1A1 (16.0%), A1A2 (52.6%), and A2A2 (31.4%). Statistically significant interactions between the *DRD2/ANKK1* TaqIA genotypes and dietary factors in relation to blood triglyceride (TG) levels were found. Carriers of the A1 allele (A1A1 homozygotes plus A1A2 heterozygotes) were protected from TG increases by maltose intake (P int. = 0.023). Instead, A2A2 homozygotes were susceptible to TG rises through consumptions of total fat (P int. = 0.041), monounsaturated fatty acids (P int. = 0.001), and dietary cholesterol (P int. = 0.019). This study suggests that the interactions between *DRD2/ANKK1* TaqIA polymorphism and dietary factors (sugar and fats) influence TG levels in diabetic patients.

## 1. Introduction

Type 2 diabetes (T2D) is a growing and alarming health problem worldwide, especially in developing countries. In 2015, the reported prevalence of T2D was 8.8% (Soto-Estrada G, 2018). In the same year, Mexico ranked sixth in the prevalence of T2D worldwide, with more than 11 million adults affected by this disease [1].

The current diet of the majority of Mexico’s population is characterized by an excessive consumption of processed foods, such as sugary beverages, sausages, and confectionary foods, as well as low intakes of fresh fruits and vegetables [2,3]. These food trends have substantially increased the proportional amounts of simple carbohydrates, saturated fatty acids, and cholesterol, with important micronutrient (vitamins and minerals) deficiencies [2,3]. Of note, these dietary patterns have been positively associated with T2D susceptibility [4,5].

Evidence suggests that genetic factors may influence food preferences and dietary intakes [6], which may contribute to the onset and progression of chronic diseases associated with nutrition, including T2D. In this context, the TaqIA polymorphism, located in the ankyrin repeat and protein kinase domain-containing protein (*ANKK1*) gene that is closely linked to the dopamine D2 receptor (*DRD2*) gene [7], has been involved in addictive behaviors toward energy-dense foods [8]. Experimental studies support that this variant affects striatal DRD2 availability [9] and specific binding potential [10]. Thus, the *DRD2/ANKK1* TaqIA polymorphism could influence individual preferences for high-fat/high-sugar foods, with a negative impact on the nutritional wellbeing.

Until now, most studies have focused on analyzing the association of genetic variants with dietary intakes, obesity features, and metabolic disorders, whereas the relevance of gene-environment interactions remains less explored, especially in T2D. In addition, these factors could differ between populations, which highlight the importance of conducting more studies that allow the early detection of T2D risk and the improvement of the therapeutic management. This study hypothesized interactions between *DRD2/ANKK1* TaqIA polymorphism and diet in relation to the metabolic status in T2D patients. The aim of this investigation was to screen relevant interactions between the *DRD2/ANKK1* TaqIA polymorphism and dietary intakes with reference to phenotypical features in patients with T2D from western Mexico.

## 2. Materials and Methods

### 2.1. Patients

This cross-sectional study enrolled 175 Mexican adults with established T2D, mainly from the states of Nayarit and Jalisco in western Mexico. Diagnosis of T2D was performed according to the American Diabetes Association (ADA) criteria [11]: Fasting plasma glucose level of 126 mg/dL (7.0 mmol/L) or higher, a 2-h plasma glucose level of 200 mg/dL (11.1 mmol/L) or higher during a 75-g oral glucose tolerance test (OGTT), or a random plasma glucose of 200 mg/dL or higher in a patient with classic symptoms of hyperglycemia, polydipsia, or polyphagia. Major exclusion criteria included a clinical history of adrenal and thyroid diseases; T2D treated with insulin; and pregnant or lactating women. The ethical principles for medical research in humans from the 2013 Helsinki Declaration were followed, and the project identification code is R-2017-1801-13 [12]. Moreover, subjects signed an informed consent to participate in this research.

### 2.2. Anthropometric Measurements

Height (cm) was measured with a stadiometer (Rochester Clinical Research, New York, NY, USA). Body weight (kg) and total body fat (%) were determined by bioelectrical impedance using the Tanita SC-331S (body composition analyzer, Tanita Corporation, Chigasaki, Japan) following the manufacturer’s instructions. Body mass index (BMI) was calculated as the ratio between body weight and squared height (kg/m^2^). Frequencies of normal weight (BMI 18.5–24.9 kg/m^2^), overweight (BMI 25.0–29.9 kg/m^2^), and obesity (BMI ≥ 30 kg/m^2^) were estimated according to the WHO criteria. Waist circumference (WC), in cm, was measured on standing subjects at the end of a natural breath, at the midpoint between the top of the iliac crest and the lower rib on the midaxillary line; whereas hip circumference (HC), in cm, was measured in the same conditions at the level of the widest circumference over the great trochanters. Both circumferences were measured by applying a stretch-resistant tape at least twice. Waist-to-hip-ratio (WHR) was estimated by dividing WC by HP.

### 2.3. Dietary Assessment

Habitual dietary intake was evaluated using 3-day food records, as detailed elsewhere [13,14,15]. Briefly, each subject was instructed to register the type, amount, and mode of preparation of all foods consumed during two weekdays and one weekend day by using food scales. The food records were reviewed and coded by a trained dietitian using the Nutritionist Pro™ Diet Analysis software (Axxya Systems, Stafford, TX, USA) in order to obtain the average daily intakes of macronutrients and micronutrients. Specific Mexican foods that were not listed in the Nutritionist Pro database were incorporated from recognized Mexican food composition tables, as previously reported [16]. Dietary reference values were based on the general recommendations by the Official Mexican Norms NOM-037-SSA2-2012 and NOM-015-SSA2-2010 of the Ministry of Health, as reported elsewhere [2,3]: Total carbohydrates, 50–60% E/day; total protein, 15% E/day; total fat, <30% E/day; saturated fatty acids (SFA) <7% E/day; monounsaturated fatty acids (MUFA) 10–15% E/d; polyunsaturated fatty acids (PUFA) 10% E/day; cholesterol, <200 mg/day; vitamin A, 900 µg/day; vitamin B1, 1.5 mg/day; vitamin B2, 1.7 mg/day; vitamin B3, 20 mg/day; vitamin B5, 10 mg/day; vitamin B6, 2 mg/day; vitamin B8, 200 µg/day; vitamin B9, 200 µg/day; vitamin B12, 2 µg/day; vitamin C, 60 mg/day; vitamin D, 400 IU/day; vitamin E, 10 IU/day; Ca, 800 mg/day; K, 1800 mg/day; Se, 55–70 µg/day; Mg, 350 mg/day; Zn, 15 mg/day.

### 2.4. Blood Tests

After an overnight fast, blood samples (10 mL) were drawn by venipuncture and centrifuged for further serum processing. Glucose, total cholesterol (TC), triglycerides (TG), and high-density lipoprotein cholesterol (HDL-c) were determined using appropriate commercial kits and read on the cobas 6000 analyzer (Roche Diagnostics International Ltd., Risch-Rotkreuz, Switzerland). Glycated hemoglobin (HbA1c) was measured with the turbidimetric immunoassay inhibition method. Low-density lipoprotein cholesterol (LDL-c) was calculated according the Friedewald equation: LDL-c = TC − (triglycerides/5) − HDL-c [17]. Also, the non-high-density lipoprotein cholesterol (non-HDL-c) was calculated as TC − HDL-c [18]. The triglyceride-glucose index (TyG index) was calculated using the formula (ln[fasting triglycerides (mg/dL) × fasting plasma glucose (mg/dL)/2]) [19]. Reference values of the biochemical tests were obtained from ATP III [20] and ADA [21] guidelines: Total cholesterol < 200 mg/dL; triglycerides < 150 mg/dL; HbA1c < 6.5%; fasting glucose < 110 mg/dL; TyG index ratio < 8.31; HDL-c > 50 mg/dL; non-HDL-c < 130 mg/dL; LDL-c < 100 mg/dL.

### 2.5. DRD2/ANKK1 Genotyping

Genomic DNA was isolated from leukocytes by a modified salting-out method [22]. Genotyping of the *DRD2/ANKK1* TaqIA polymorphism was performed by a polymerase chain reaction-restriction fragment length polymorphism (PCR-RFLP) assay [23]. The accuracy of the *DRD2/ANKK1* genotyping was verified using positive controls for the three possible genotypes (A1A1, A1A2, A2A2) and a negative template control in each experiment. Moreover, 25% of the total samples were re-genotyped, and of these, 100% were concordant. The Hardy–Weinberg equilibrium (HWE) was assessed using the Convert 1.31 and the Arlequin 3.0 software. In order to corroborate the homogeneity of the sample, the analysis of molecular variance (AMOVA) was performed in the Arlequin software 3.0, as reported elsewhere [24].

### 2.6. Statistical Analyses

The distribution of the study variables (normality) was evaluated using the Kolmogorov–Smirnov test. Anthropometric, metabolic, and dietary variables were normally distributed according to a *p* value > 0.05 in all Kolmogorov-Smirnov tests. Thus, parametric statistical tests were used. Continuous variables were reported as means ± standard deviations, whereas categorical variables were expressed as numbers and percentages. Genotype comparisons were performed as follows: A1 carriers (A1A1 and A1A2 genotypes) versus A2A2 homozygotes. Statistical differences in quantitative and categorical variables between the *DRD2/ANKK1* genotypes were estimated by Student’s *t*-test and chi-square test, respectively. Relevant gene-diet interactions were calculated with multiple linear regression tests adjusted by energy intake, age, sex, and BMI. Statistical analyses were performed in the statistical programs Stata 12 (StataCorp LLC, College Station, TX, USA; www.stata.com) and IBM SPSS 20 (IBM Inc., Armonk, NY, USA). Statistical significance was set at *p* < 0.05.

## 3. Results

### 3.1. Distribution of the DRD2/ANKK1 TaqIA Polymorphism and Characteristics of the Study Population

Genotype frequencies of the DRD2/ANKK1 TaqIA polymorphism were A1A1 (16.0%), A1A2 (52.6%), and A2A2 (31.4%), whereas the frequencies of the A1 and A2 alleles were 42.3% and 57.7%, respectively. The distribution of the DRD2/ANKK1 genotypes was concordant with the HWE (*p* = 0.45). Also, the population was genetically homogeneous regarding this genetic variant, as revealed by AMOVA analyses (*p* = 0.32).

Demographic, anthropometric, and clinical characteristics of the diabetic patients by DRD2/ANKK1 genotypes are reported in Table 1. Overall, nearly 77% of the total sample had excessive adiposity according to BMI cutoffs. No statistically significant differences in age, sex, or body composition markers between A1 carriers and A2A2 homozygotes were found. Also, both genotype groups underwent a similar time of T2D evolution.

### 3.2. Daily Dietary Intake by Genotyes of the DRD2/ANKK1 TaqIA Polymorphism

The average daily intakes of macronutrients and micronutrients by *DRD2/ANKK1* genotypes are shown (Table 2, Table 3, respectively). On the one hand, all subjects consumed protein, total fat, saturated fatty acids (SFA), and dietary cholesterol above the recommendations for the general population (Table 2). On the other hand, lower intakes of fiber and polyunsaturated fatty acids (PUFA) than the reference values were observed regardless of the *DRD2/ANKK1* genetic profile (Table 2). Overall, deficient intakes of most vitamin B complex (B1, B2, B3, B5, and B6) as well as vitamins D and E, calcium, magnesium, and zinc were also found (Table 3).

### 3.3. Biochemical Profile by Genotyes of the DRD2/ANKK1 TaqIA Polymorphism

The biochemical profile of the diabetic patients by *DRD2/ANKK1* genotypes are reported in Table 4. As expected, glycemic traits (fasting glucose and HbA1c) were within the established parameters for the diagnosis of diabetes. Moreover, blood concentrations of TG and non-HDL-c as well as the TyG index were above the reference values. There were no relevant relationships between DRD2/ANKK1 genotypes and the studied blood markers.

### 3.4. DRD2/ANKK1-Diet Interactions

**Figure 1:**
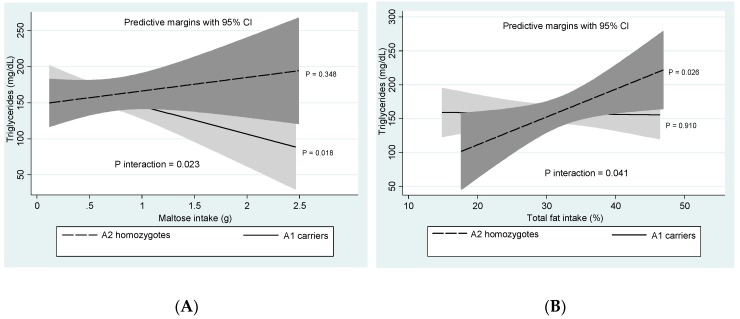

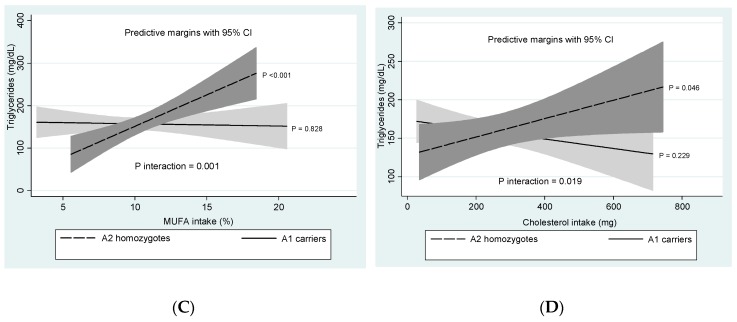
*DRD2/ANKK1*-diet interactions with reference to blood triglyceride levels. (**A**) *DRD2/ANKK1*-maltose intake interaction. (**B**) *DRD2/ANKK1*-fat intake interaction. (**C**) *DRD2/ANKK1*-MUFA intake interaction. (**D**) *DRD2/ANKK1*-cholesterol intake interaction. MUFA: Monounsaturated fatty acids.

Statistically significant *DRD2/ANKK1*-diet interactions concerning blood triglyceride levels are plotted in Figure 1. Carriers of the A1 allele (A1A1 homozygotes plus A1A2 heterozygotes) were protected from TG increases by maltose intake (P int. = 0.023) (Panel A). Instead, A2A2 homozygotes were genetically susceptible to TG rises after the increasing consumptions of total fat (P int. = 0.041), monounsaturated fatty acids (MUFA) (P int. = 0.001), and dietary cholesterol (P int. = 0.019) (Panels B, C, and D, respectively). No relevant gene-diet interactions regarding body composition and other metabolic phenotypes were found.

## 4. Discussion

Globally, the pattern of distribution of the *DRD2/ANKK1* TaqIA polymorphism present important variations, especially among populations exposed to different environments [25]. In this investigation, about 42% of the studied Mexican population carried the A1 allele. Comparable frequencies have been reported in mestizo free-diabetes subjects living at the same West Mexican regions, including Nayarit (51%) and Jalisco (47%) [26]. To date, Native groups from Mexico (Nahuas, Huicholes or Wixárika, Mayas, and Pima Indians) exhibited one of the highest frequencies (>60%) of this allele worldwide, which is related to specific ancient and long-lasting cultural/lifestyle factors [26,27,28]. Conversely, European countries have the lowest prevalence of this genetic profile (nearly 20%), whereas Asian and African reference populations present a frequency of approximately 40% according to international genome consortiums [29,30].

Mixed evidence suggests that genetic variations in dopamine genes may disrupt the energy/pleasure neurocircuitry, with implications in food intake and eating behaviors [31,32]. In this study, no differences in dietary intakes by *DRD2/ANKK1* genotypes were found. This finding may be related to the fact that all studied patients are immersed in the same obesogenic environment, which has been reported to be characterized by the overconsumption of high-fat/sugar foods, such as over-fried foods cooked in oil or lard, red meat, and pastry products [33]. This Westernized dietary pattern may negatively impact not only diabetes incidence, but also disease risk progression and the medical treatment outcomes [34]. In another study, black diabetic participants carrying the *DRD2/ANKK1* A1 allele had significantly greater intake of total fat, saturated fat, and cholesterol compared with A2A2 homozygote individuals; however, these results were not replicated in diabetic patients with European ancestry [35].

Interactions between genetic and environmental factors may also influence dietary intakes and related disease risk [36], and may help to explain, at least in part, some of the inconsistencies among observational studies by associating genetic variation with nutritional and obesity features. In this research, apparently for the first time, relevant interactions between the *DRD2/ANKK1* TaqIA polymorphism and nutrient intakes regarding blood TG levels were found. This result is consistent with our hypothesis that interactions between *DRD2/ANKK1* TaqIA polymorphism and diet could impact the metabolic status. Interestingly, differential genotype effects were detected depending on the nutritional profiles. Thus, increasing maltose intake was not a risk factor for TG rises in A1 allele carriers. Instead, participants carrying the A2/A2 genotype whose fat intake (total fat and MUFA) was low, seemed to present lower TG levels, whereas an opposite effect was found in higher fat intake participants. This finding highlights the effect of dietary fat on the blood lipid profile, where the *DRD2/ANKK1* genotype may play an indirect role. In addition, other genetic (lipid-related variants) and non-genetic factors (individual preferences, type of food, physical activity, and insulin resistance level) may influence the high-fat intake and increased TG levels. These results are consistent with the influence of the *DRD2/ANKK1* TaqIA polymorphism on food preferences and the related metabolic phenotype. In this context, glucose and lipid abnormalities were common among TaqI A1 allele carriers, who consumed unhealthier foods than the rest of genotype groups in a general Mexican population [37]. Moreover, an increase in calorie intake from sugar and adiposity was observed in a multi-ethnic sample of children with two TaqI A1 alleles than non-A1 allele carriers [38]. Additionally, a potential interaction between variations in *DRD2/ANKK1* (rs1800497, TaqI A1 allele carriers) and *FTO* (rs8050136, A allele carriers) genes was associated with increased body fat and reduced peripheral insulin sensitivity in a German family study [39].

The limitations of this study include a relatively small sample, and the inherent bias of self-declaration on food intake and the analysis of the current diet. Also, due to the cross-sectional design of this study, no causality between the *DRD2/ANKK1* genetic variant and food intake as well as metabolism phenotype, could be established. Therefore, further studies with a greater number of individuals, a long-term diet evaluation, and a causality approach are required.

Hypertriglyceridemia remain as one the most frequent forms of dyslipidemia among the Mexican population, and also in diabetic patients [40]. High serum levels of TG are implicated in insulin resistance and represent an important risk factor for T2D and liver cirrhosis in susceptible individuals, which are the leading causes of morbidity and mortality in the country [41]. Lipid disorders are determined by genetic and environmental/lifestyle factors [42]. Given the effect of the *DRD2/ANKK1* TaqIA polymorphism on dietary intakes and metabolic status, the detection of this genotype along with other nutrient-sense genes could be an auxiliary tool for the identification of high-risk groups, as well as for a precise nutrition management of hypertriglyceridemia in T2D and the prediction of resistance or responsiveness to dietary interventions.

## 5. Conclusions

In conclusion, this study suggests that the interactions between *DRD2/ANKK1* TaqIA polymorphism and dietary factors (mainly maltose, total fat, and MUFA) influence TG blood levels in patients with T2D. This information could be useful for the identification of high-risk groups, as well as for the precision nutrition management of hypertriglyceridemia in T2D.

## Figures and Tables

**Table 1:** Characteristics of the diabetic patients according to genotypes of the *DRD2/ANKK1* TaqIA polymorphism.

Variable	A1 Carriers *n* = 120	A2 Homozygotes *n* = 55	*p* Value
Age (years)	58.4 ± 11.0	57.7 ± 11.1	0.699
Sex (F/M)	77/43	34/21	0.866
Years with T2D	7.7 ± 8.79	7.7 ± 7.54	0.847
BMI (kg/m^2^)	29.8 ± 5.7	29.5 ± 5.2	0.711
Normal weight, *n* (%)	21 (18.4%)	12 (22.6%)	0.804
Overweight, *n* (%)	45 (39.4%)	20 (37.7%)	
Obesity, *n* (%)	48 (42.1%)	21 (39.6%)	
WHR	0.95 ± 0.104	0.94 ± 0.074	0.405
Total body fat %	33.8 ± 8.55	34.0 ± 7.78	0.894

Values are presented as means ± standard deviations. F: females; M: males; T2D: Type 2 diabetes; BMI: Body mass index; WHR: Waist-to-hip ratio.

**Table 2:** Macronutrient intakes according to genotypes of the *DRD2/ANKK1* TaqIA polymorphism.

Nutrient	Reference Values	A1 Carriers	A2 Homozygotes	*p* Value
Total energy (Kcal/d)	-	1556.8 ± 401.7	1554.7 ± 450.8	0.975
Total carbohydrates (%E/d)	50–60	50.6 ± 10.2	47.9 ± 9.8	0.091
Glucose (g/d)	-	10.8 ± 9.3	9.7 ± 6.8	0.457
Galactose (g/d)	-	0.05 ± 0.27	0.02 ± 0.07	0.459
Fructose (g/d)	-	14.85 ± 11.82	13.4 ± 10.1	0.442
Sacarose (g/d)	-	10.8 ± 8.1	8.9 ± 6.9	0.120
Lactose (g/d)	-	1.2 ± 3.5	1.2 ± 3.0	0.924
Maltose (g/d)	-	0.68 ± 0.42	0.73 ± 0.56	0.486
Sugar (g/d)	-	54.5 ± 31.6	50.7 ± 24.5	0.440
Fiber (g/d)	30	20.8 ± 6.9	18.7 ± 6.4	0.060
Total protein (%E/d)	15	17.7 ± 3.1	18.0 ± 2.8	0.657
Total fat (%E/d)	<30	30.7 ± 6.6	32.2 ± 6.0	0.178
SFA (%E/d)	<7	8.4 ± 2.6	8.5 ± 2.4	0.838
MUFA (%E/d)	10–15	10.0 ± 2.8	10.7 ± 2.7	0.137
PUFA (%E/d)	10	7.6 ± 2.7	8.1 ± 2.4	0.221
Cholesterol (mg/d)	<200	264.2 ± 136.1	280.6 ± 184.4	0.511

Values are presented as means ± standard deviations. SFA: saturated fatty acids; MUFA: monounsaturated fatty acids; PUFA: polyunsaturated fatty acids; d: day.

**Table 3:** Micronutrient intakes according to genotypes of the *DRD2/ANKK1* TaqIA polymorphism.

Nutrient	Reference Values	A1 Carriers	A2 Homozygotes	*p* Value
Vitamin A (µg/d)	900	389.7 ± 247.0	301.4 ± 194.4	0.020
Vitamin B1 (mg/d)	1.5	1.02 ± 0.35	1.02 ± 0.33	0.891
Vitamin B2 (mg/d)	1.7	1.29 ± 0.48	1.26 ± 0.46	0.701
Vitamin B3 (mg/d)	20	15.8 ± 5.8	15.7 ± 5.3	0.888
Vitamin B5 (mg/d)	10	3.55 ± 1.86	3.31 ± 1.59	0.404
Vitamin B6 (mg/d)	2	1.79 ± 0.73	1.68 ± 0.59	0.303
Vitamin B8 (µg/d)	200	8.29 ± 5.98	8.24 ± 6.66	0.963
Vitamin B9 (µg/d)	200	255.3 ± 118.4	228.3 ± 82.4	0.128
Vitamin B12 (µg/d)	2	3.05 ± 3.76	2.52 ± 1.33	0.306
Vitamin C (mg/d)	60	83.0 ± 75.9	72.3 ± 83.7	0.402
Vitamin D (IU/d)	400	136.4 ± 83.4	139.7 ± 95.2	0.820
Vitamin E (IU/d)	10	5.96 ± 5.90	6.05 ± 5.60	0.924
Ca (mg/d)	800	609.9 ± 238.4	591.4 ± 300.9	0.661
K (mg/d)	1800	2503.6 ± 656.7	2353.9 ± 765.1	0.186
Se (µg/d)	55–70	68.9 ± 27.0	71.2 ± 29.0	0.615
Mg (mg/d)	350	295.9 ± 85.5	286.2 ± 91.4	0.499
Zn (mg/d)	15	8.57 ± 3.99	8.5 ± 2.7	0.867

d: day; Values are presented as means ± standard deviations. Bold numbers indicate *p* < 0.05.

**Table 4:** Biochemical profile according to genotypes of the *DRD2/ANKK1* TaqIA polymorphism.

Biochemical Variable	Reference Values	A1 Carriers	A2 Homozygotes	*p* Value
Total cholesterol (mg/dL)	<200	179.5 ± 34.5	178.5 ± 31.5	0.801
Triglycerides (mg/dL)	<150	157.3 ± 75.7	161.3 ± 81.6	0.891
HbA1c (%)	<6.5	7.50 ± 2.00	7.51 ± 1.96	0.701
Glucose (mg/dL)	<110	144 ± 57.2	152 ± 70.3	0.421
TyG index (ratio)	<8.31	9.14 ± 0.62	9.32 ± 0.78	0.097
HDL-c (mg/dL)	>50	46 ± 12.1	49 ± 14.8	0.315
Non-HDL-c (mg/dL)	<130	135 ± 34.5	130 ± 29.2	0.349
LDL-c (mg/dL)	<100	104 ± 30.3	98 ± 24.5	0.214

Values are presented as means ± standard deviations. HbA1c: Glycated haemoglobin; TyG index: Triglyceride-glucose index; HDL-c: High-density lipoprotein cholesterol; Non-HDL-c: Non-high-density lipoprotein cholesterol; LDL-c: Low-density lipoprotein cholesterol.
